# Site-Differentiated Iron–Sulfur Cluster Ligation Affects Flavin-Based Electron Bifurcation Activity

**DOI:** 10.3390/metabo12090823

**Published:** 2022-09-01

**Authors:** Courtney E. Wise, Anastasia E. Ledinina, Carolyn E. Lubner

**Affiliations:** Biosciences Center, National Renewable Energy Laboratory, Golden, CO 80401, USA

**Keywords:** electron bifurcation, biochemistry, iron–sulfur cluster, flavoenzyme, energy conservation, bioenergetics, biological electron transfer, thermodynamics, metabolism

## Abstract

Electron bifurcation is an elegant mechanism of biological energy conversion that effectively couples three different physiologically relevant substrates. As such, enzymes that perform this function often play critical roles in modulating cellular redox metabolism. One such enzyme is NADH-dependent reduced-ferredoxin: NADP^+^ oxidoreductase (NfnSL), which couples the thermodynamically favorable reduction of NAD^+^ to drive the unfavorable reduction of ferredoxin from NADPH. The interaction of NfnSL with its substrates is constrained to strict stoichiometric conditions, which ensures minimal energy losses from non-productive intramolecular electron transfer reactions. However, the determinants for this are not well understood. One curious feature of NfnSL is that both initial acceptors of bifurcated electrons are unique iron–sulfur (FeS) clusters containing one non-cysteinyl ligand each. The biochemical impact and mechanistic roles of site-differentiated FeS ligands are enigmatic, despite their incidence in many redox active enzymes. Herein, we describe the biochemical study of wild-type NfnSL and a variant in which one of the site-differentiated ligands has been replaced with a cysteine. Results of dye-based steady-state kinetics experiments, substrate-binding measurements, biochemical activity assays, and assessments of electron distribution across the enzyme indicate that this site-differentiated ligand in NfnSL plays a role in maintaining fidelity of the coordinated reactions performed by the two electron transfer pathways. Given the commonality of these cofactors, our findings have broad implications beyond electron bifurcation and mechanistic biochemistry and may inform on means of modulating the redox balance of the cell for targeted metabolic engineering approaches.

## 1. Introduction

Flavin-based electron bifurcation (FBEB) is a recently discovered mechanism of energy coupling in microbes that enables a number of challenging chemical reactions [1]. FBEB allows for a thermodynamically unfavorable process to be driven by direct coupling to a net exergonic reaction. This mechanism is utilized in nitrogen fixation, proton translocation, H_2_ metabolism, and the Wood–Ljungdahl pathway for CO_2_ fixation, as well as in the Clostridial pathway for *n*-butanol production and the “chain elongation” reaction for production of C4-C8 compounds from ethanol and acetate, among others [2,3,4]. Many microbial systems investigated for industrial applications are believed to rely on bifurcating enzymes.

(1) $${\mathrm{NAD}}^{+}{+\;2\;\mathrm{NADPH}\;+\;2\;\mathrm{Fd}}_{\;\mathrm{Ox}}⇌{\;2\;\mathrm{NADP}}^{+}+2\;{\mathrm{Fd}}_{\;\mathrm{Red}}+H^{+}+\mathrm{NADH}$$

(2) $$\mathrm{NADH}+2\;{\mathrm{NADP}}^{+}+\;2\;{\mathrm{Fd}}_{\;\mathrm{Red}}{\;+H}^{+}⇌{\;2\;\mathrm{NADPH}+2\;\mathrm{Fd}}_{\;\mathrm{Ox}}+{\mathrm{NAD}}^{+}$$

FBEB systems utilize the oxidation of an initiating substrate to facilitate the reduction of two other substrates, thus playing a significant role in the modulation of intracellular reducing equivalents. Indeed, deletion mutants of FBEB enzymes from multiple organisms have been demonstrated to disrupt energy distribution, reduce fermentation performance, and impact cell viability [5]. This makes FBEB an attractive target to modify for altering the flux of energy through metabolic pathways. Despite this, the understanding of how the protein environment facilitates this exquisite reactivity, and thus the ability to rationally manipulate the biochemical features of these enzymes, is currently lacking.

**Figure 1:**
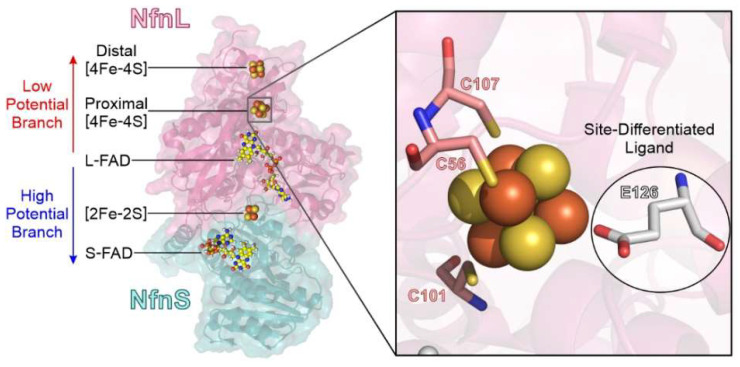
NfnSL is a heterodimer composed of a small (NfnS, in teal) and a large (NfnL, in pink) subunit (PDB 5JCA [10]). Upon oxidation of NADPH by L-FAD, the first bifurcated electron is transferred to the high-potential catalytic branch (blue arrow) of NfnS, comprised of a [2Fe-2S] cluster and S-FAD, for reduction of NAD^+^. The second bifurcated electron traverses the proximal and distal [4Fe-4S] clusters of the low-potential branch (red arrow) housed within the NfnL subunit for reduction of Fd. The proximal cluster of NfnL (right) features a site-differentiated glutamate ligand at amino acid position 126 (white), which contrasts with the canonical cysteine linkage (pink) of many FeS cofactors. In this work, NfnL-E126 was mutated to a Cys residue to allow for study of the impact of atypical FeS ligation upon NfnSL biochemistry.

Several FBEB enzymes are well-characterized both mechanistically and structurally [1,6,7,8], which lays a foundation for exploring the influence of key structural features on enzyme biochemical activity. Here, we focus on an unusual element of an iron–sulfur (FeS) cluster cofactor found within the NADH-dependent reduced-ferredoxin (Fd):NADP^+^ oxidoreductase FBEB enzyme NfnSL (Figure 1). NfnSL is a heterodimeric protein that contains several electron transferring cofactors along two bifurcated pathways. These branches work in concert to catalyze the reduction of Fd and NAD^+^ from NADPH in what is referred to as the bifurcation reaction (Equation (1)), as well as the reverse reaction (the oxidation of Fd and NADH to form NADPH) which is termed confurcation (Equation (2)). Because NfnSL links three important intracellular redox pools (NADH, NADPH, and Fd), it likely has a critical role in redox balancing in vivo. Central to the two pathways is a flavin cofactor, L-FAD, with properties tuned for transferring two electrons at different reduction potentials individually along each of the catalytic branches [9]. The bifurcating flavin in NfnSL possesses a low reduction potential for the oxidized (Ox) to fully reduced hydroquinone (HQ) two-electron couple [9] and a characteristic inverted potential arrangement [10], where the one-electron reduced anionic semiquinone (ASQ) is thermodynamically destabilized and short-lived [1]. Because of these features, the individual bifurcated electron transfer events are constrained to specific thermodynamic and kinetic regimes [1,8,11]. Interestingly, the initial acceptors of these bifurcated electrons are FeS clusters, each displaying a unique and non-canonical amino acid ligand in which an aspartate or glutamate replaces a cysteine residue. Site-differentiated FeS clusters are commonly found within redox active enzymes and have been shown to affect cofactor geometry, reduction potential, electronic properties, and/or electron transfer; however, there is no clear consensus for how a particular ligand may change these parameters [12,13,14,15,16,17,18,19]. Due to their prominent placement within Nfn, we hypothesize that site-differentiated ligation is an integral feature for coupling with L-FAD to ensure the proper partitioning of electrons among the high- and low-potential branches. 

**Figure 2:**
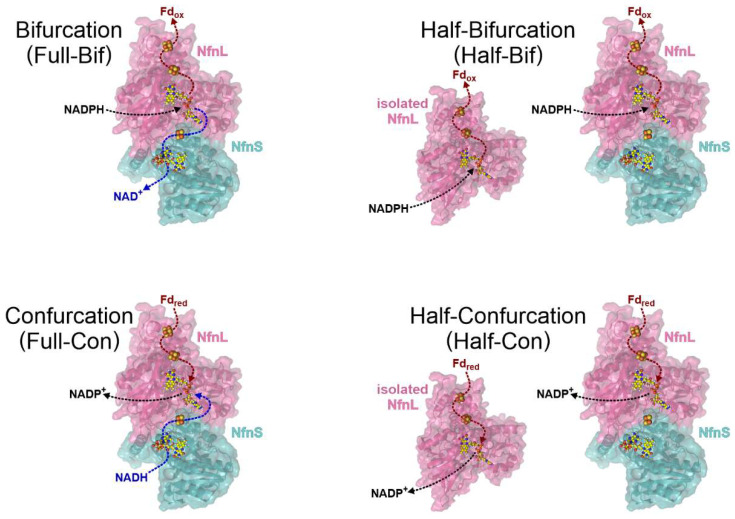
Schemes of the biochemical profiles probed in this work, where full reactions involve both catalytic branches, and half-reactions involve only the low-potential branch. Full bifurcation (**top left**) is the oxidation of NADPH leading to the reduction of both NAD^+^ and Fd. Half-bifurcation (**top right**) is described as the reduction of Fd only from NADPH. Full confurcation (**bottom left**) is the oxidation of Fd and NADH leading to the reduction of NADP^+^. Half-confurcation (**bottom right**) is described as the oxidation of Fd only for the reduction of NADP^+^. The half-reactions were assessed in NfnL (WT or E126C) both with and without NfnS (PDB 5JCA [10]).

In this study, we demonstrate that site-differentiation of the proximal [4Fe-4S] cluster along the low-potential branch in the NfnL subunit plays a key role in ensuring that electron transfer along the two pathways is coupled. Biochemical profiles of wild-type (WT) NfnSL and a variant in which the [4Fe-4S] cluster Glu-ligand was substituted by Cys (NfnL-E126C) were assessed for the isolated NfnL subunits as well as the holoenzymes (i.e., NfnSL-WT and NfnSL-E126C). Mutation of this single amino acid resulted in significant disruption in electron transfer along the low-potential branch. This was most apparent when probing the catalysis of the single branch reactions (i.e., half-bifurcation, red in Equation (1), or half-confurcation, blue in Equation (2); Figure 2). Furthermore, alteration of this ligand additionally affected the accumulation of electrons along the high-potential branch over ~35 Å away from the altered cofactor. Our results demonstrate that FeS ligation contributes to the coupling and control of electron distribution in FBEB and adds to the repertoire of effects that non-canonical ligands exert upon cofactors. The knowledge generated here is expected to provide insight into not only the mechanisms of FBEB, but also how features of the protein environment favor integration with three different physiological substrate pools. 

## 2. Materials and Methods

### 2.1. Plasmid Construction

The *nfnS* gene from *Pyrococcus furiosus* DSM 3638 that encodes the small NfnS subunit (accession number AAL81452) was codon-optimized and synthesized for expression in *Escherichia coli* by GenScript, USA. A strep-tag with protein sequence GWSHPQFEK followed by a stop codon, with the corresponding DNA sequence GGCTGGAGCCACCCGCAGTTCGAGAAATAA, was included at the C-terminal end of the synthesized *nfnS* construct in a pUC57 backbone. This gene was then subcloned into pETDuet^TM^-1 (Novagen, Burlington, MA, USA) using NcoI (CCATGG) and BamHI (GGATCC) restriction sites. The *fd* gene (accession number NZ_CP023154) from *P. furiosus* DSM 3638 was also codon-optimized for expression in *E. coli* with a strep-tag (GWSHPQFEKGS and encoded by DNA sequence GGATGGTCACACCCCCAATTTGAAAAAGGCAGC) inserted immediately after the start codon for the gene. The synthesized construct was placed into pETDuet^TM^-1 between the NcoI (CCATGG) and BamHI (GGATCC) restriction sites. The construct for expression of NfnL-E126C resulted from site-directed mutagenesis of the glutamate at the 126 position to a cysteine, using the NfnL plasmid described in [9] as the template. All plasmids constructed were verified by commercial sequencing (GenScript, Piscataway, NJ, USA). 

### 2.2. NfnSL Protein Overexpression and Purification

Expression and purification of the NfnL-WT, NfnL-E126C, and NfnS was carried out as previously described [9]. Heterologous expression of Fd was carried out in *E. coli* BL21(DE3) cells. A 1:100 volume of overnight starter culture was used to inoculate 1 L volumes of terrific broth in 2 L flasks which also contained trace metals, 0.4% glycerol, 125 µg·mL^−1^ thiamine-HCl, and 100 µg·mL^−1^ carbenicillin. Cultures were incubated at 37 °C while shaking at 250 rpm until an optical density at 600 nm of ~1.2 was achieved, at which point ferric ammonium citrate was added to a final concentration of 2.5 mM and expression was induced using 1.0 mM isopropyl β-d-1-thiogalactopyranoside. Growth was continued at 37 °C and 250 rpm for ~60 min, after which 2 L of culture were combined into a single septum-sealed 2 L flask and supplanted with 10 mM sodium fumarate and 0.5% *w*/*v* d-glucose. Following a static 30 min incubation at room temperature, l-cysteine was added to a concentration of 1 mM and cultures were sparged with argon gas at room temperature overnight. Cultures were harvested anaerobically by centrifugation, then cell pellets were resuspended in 50 mM HEPES pH 8.8 with 20 mM NaCl, 5% glycerol, and 2 mM DTT and stored in septum-sealed vials at −80 °C.

Cell lysis and purification of all proteins was performed using established protocols [9] with the following modifications. Cell lysis was carried out via sonication in an mBraun anaerobic chamber. Following ultracentrifugation, clarified lysate was transferred onto a gravity column containing high-capacity Strep-Tactin XT-4Flow resin (IBA Life Sciences, Göttingen, Germany). Resin was equilibrated with 10 column volumes (CV) of either 150 mM HEPES pH 8.8 with 200 mM NaCl and 5% glycerol (*Nfn buffer*) for purification of the NfnL-WT, NfnL-E126C, and NfnS subunits or with 50 mM HEPES pH 8.8 with 20 mM NaCl and 5% glycerol (*Fd buffer*) for Fd. Bound protein was washed with 2–5 CV of Nfn or Fd buffer and eluted with the same buffer containing 25 mM d-biotin. Biotin, which absorbs at 260 nm and interferes with protein quantification using the 280 nm absorbance, was then removed from the solution by sequential wash cycles using an Amicon stirred cell with a 5 kDa MWCO filter. After this buffer exchange into Fd buffer, purified Fd was quantified spectroscopically at 280 nm [20] (for apo-Fd) and 390 nm [21] (for holo-Fd) using extinction coefficients of 13 and 17 mM^−1^ cm^−1^, respectively. NfnL-E126C, NfnS, and Fd demonstrated soluble post-purification yields of 25, 150, and 5 mg of protein per L culture, respectively. SDS-PAGE showed >90% purity for NfnL-E126C and NfnS and revealed that Fd was ~85% free of contaminant protein. Iron–sulfur (FeS) content was assessed based on an A_390_/A_280_ ratio of 0.57 for cofactor-replete Fd [22].

### 2.3. Cofactor Reconstitution and Protein Preparation

Reconstitution of NfnL-WT and NfnL-E126C, along with iron and flavin quantification for the NfnL-WT, NfnL-E126C, and NfnS subunits, was performed using previously described methodologies [9]. NfnS reconstitution was carried out anaerobically in pH 8.8 Nfn buffer at a protein concentration of 100 µM. After β-mercaptoethanol was added to 1% (*v*/*v*) while stirring, FAD and NAD^+^ were added to 200 and 400 µM, respectively. The reaction was incubated for 30 min at room temperature, then ferrous ammonium sulfate was added slowly over 5 min to 800 µM. Following a 15 min incubation, sodium sulfide was introduced in the same manner to 800 µM, then the reaction was septum-sealed and heated at 42 °C overnight. Excess material was removed by repeated washing via Amicon stirred cell equipped with a 10 kDa cutoff filter until filtrate ran clear, then cofactor quantification was performed in the manner detailed for NfnL and NfnSL. 

After purification, Fd was found to lack full incorporation of its single [4Fe-4S] cluster. Reactions containing 100 µM enzyme in buffer with 1% β-mercaptoethanol were incubated for 30 min at room temperature while stirring. Ferrous ammonium sulfate was slowly added over 5 min while stirring to a final concentration of 600 µM. After 15 min, sodium sulfide was added in the same quantity and manner as iron. The reaction was incubated overnight at room temperature. Excess material was removed from Fd by desalting the reconstitution reactions using PD-10 desalting columns (GE Life Sciences) into buffer. Reconstituted protein was centrifuged to eliminate any precipitated material, then iron incorporation was assessed via UV-visible spectroscopy. For full iron incorporation the absorbance ratio of A_390_/A_280_ is 0.570 [22]. All reconstitution steps were carried out in an anaerobic chamber.

Reduced Fd was generated by incubating 300 µM protein with 300 µM sodium dithionite at room temperature for 1 h in an anaerobic chamber. Dithionite was then removed from the solution using a PD Miditrap G-25 column desalting column from GE healthcare. The reduced state of Fd was confirmed via UV-vis spectroscopy at 412 nm (ɛ_oxidized_ = 16 mM^−1^ cm^−1^ and ɛ_reduced_ = 9 mM^−1^ cm^−1^). When fully reduced, the absorbance at 412 nm is 55% of the absorbance of the oxidized ferredoxin [22].

### 2.4. Biochemical Characterizations

Fluorescence binding titrations for NADP(H) with NfnL-E126C were carried out as described for NfnL-WT in [9]. Dye-based steady-state kinetics experiments were also performed according to established protocols [9], except using 10 µM of enzyme (NfnL-WT or NfnL-E126C, with and without NfnS).

Bifurcation and confurcation activity assays differ in the oxidative states of the substrates present, and therefore in the overall directionality of the reaction, as shown in Figure 2. All activity assays were carried out anaerobically and at room temperature in pH 8.8 Fd buffer, using 1 µM of NfnL-WT, NfnL-E126C, NfnS with NfnL-WT, or NfnS with NfnL-E126C enzyme(s). Pyridine nucleotide stocks were prepared fresh prior to use and quantified spectroscopically using ε_260nm_ = 17.5 mM^−1^·cm^−1^ for NAD(P)^+^ and ε_340nm_ = 6.22 mM^−1^·cm^−1^ for NAD(P)H. Full-bifurcation reactions, performed with NfnSL-WT and NfnSL-E126C, were initiated by addition of the NADPH, NAD^+^, and oxidized Fd substrates to a final concentration of 50 µM. Full-confurcation reactions were carried out in the same manner, but using NADP^+^, NADH, and reduced Fd as initiating substrates. Half-bifurcation and half-confurcation reactions omitted NAD^+^ and NADH, respectively, and were performed with NfnL-WT and NfnL-E126C both as isolated monomers as well as in complex with NfnS. Reactions were carried out in 100 µL volumes in a septum-sealed quartz cuvette with a 1 cm pathlength. Activity, expressed in units (U; defined as µmol substrate per minute) per mg Nfn, was monitored based on the reduction (for bifurcation) or oxidation (confurcation) of Fd at 418 nm using the kinetics module of a CARY 4000 UV-visible spectrophotometer with an averaging time of 0.1 s for 3 to 5 min.

Experiments seeking to assess the accumulation of electrons along the high-potential catalytic branch housed within the NfnS subunit in the absence of NAD(H) were carried out anaerobically in low-volume septum-sealed quartz cuvettes with a 0.3 cm pathlength. Reactions containing 10 µM NfnSL-WT or NfnSL-E126C, both with and without 10 µM oxidized Fd, along with increasing concentrations of NADPH from 0 to 15 µM in pH 7.0 Nfn buffer, were allowed to reach equilibration in an anaerobic chamber at room temperature prior to spectroscopic analysis via a Cary 4000 spectrophotometer. The spectra collected for these samples were then analyzed for increases in absorbance ~610 nm corresponding to increasing concentrations of NADPH. The presence of such optical features is indicative of neutral semiquinone (NSQ) formation at the S-FAD cofactor [10], and thus can be compared between NfnSL-WT and NfnSL-E126C to assess for alterations in electron distribution along the NfnS pathway at limiting concentrations of NADPH. 

All data were processed using OriginPro software, after which publication-quality figures were generated with Corel Designer.

## 3. Results

### 3.1. Protein Expression, Purification, and Reconstitution

NfnL-E126C, NfnS, and Fd were heterologously expressed in *E. coli* and following reconstitution of FeS and flavin, cofactor quantification was performed to assess incorporation. Mutation of one of the ligands, E126, to the proximal FeS cluster of NfnL, did not appear to impact iron–sulfur cluster binding, with counting assays revealing 9.1 ± 0.9 iron atoms (~115% Fe incorporation) and 1.3 ± 0.2 FAD (130% flavin-loading) per NfnL-E126C. Each mole of NfnS was found to contain 2.4 ± 0.1 iron atoms (120% Fe incorporation) and 0.85 ± 0.03 FAD (85%). Spectroscopic analysis of Fd showed 67% FeS incorporation, with the maximum absorbance in the visible region at ~390 nm [22]. The concentration of Fd used following reconstitution is based on the FeS-replete protein, which is quantified from the 390 nm absorbance as described in the Methods.

### 3.2. Dye-Based Steady-State Kinetics

Dye-based steady-state kinetics experiments were employed to assess the impact of the E126C mutation on the ability of NfnL, both in the presence and absence of NfnS, to facilitate the reduction of benzyl viologen using electrons from NADPH (Table 1). The isolated NfnL subunit yielded *K*_M_ values of 1.3 ± 0.1 mM for NfnL-WT and 0.30 ± 0.03 mM for NfnL-E126C. The *K*_M_ for NfnSL-WT and NfnSL-E126C were remarkably similar, at 0.37 ± 0.02 mM and 0.37 ± 0.01, respectively. Catalytic efficiency, calculated by taking the ratio of *k*_cat_ to *K*_M_, was found to be 1.7 ± 0.3 mM^−1^·s^−1^ for NfnL-WT, 12 ± 3 mM^−1^·s^−1^ for NfnL-E126C, 15 ± 2 mM^−1^·s^−1^ for NfnSL-WT, and 16 ± 2 mM^−1^·s^−1^ for NfnSL-E126C. No significant difference in *K*_M_, *V*_max_, *k*_cat_, or catalytic efficiency was observed between NfnSL-WT and NfnSL-E126C. In the absence of NfnS, however, the two isolated versions of NfnL diverged appreciably. NfnL-WT exhibited the highest *K*_M_ of the proteins assayed here, along with the lowest values of *V*_max_, *k*_cat_, and catalytic efficiency, demonstrating a significantly inhibited ability to reduce the benzyl viologen dye. Isolated NfnL-E126C demonstrated the lowest *K*_M_, with values of *V*_max_ and *k*_cat_ higher than those of NfnL-WT (but still lower than either NfnSL version) and a catalytic efficiency comparable to NfnSL. 

### 3.3. Binding of NADP(H) to NfnL-E126C

**Figure 3:**
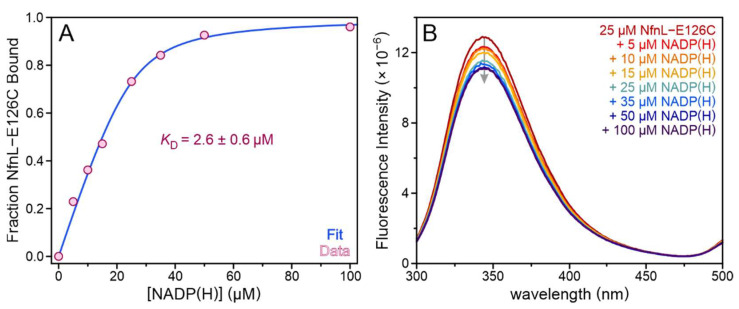
Fluorescence binding titrations of NfnL-E126C with NADP(H) revealed a *K*_D_ of 2.6 ± 0.6 µM and no significant difference in binding affinity for the reduced versus the oxidized form of this substrate, as shown in the representative data (**A**). Following excitation of aromatic residues within the protein at 285 nm, a decrease in fluorescence emission was observed at 345 nm that corresponded to increasing concentrations of NADP(H) (**B**). These results are comparable to the previously reported *K*_D_ of 3.0 ± 0.4 µM for WT NfnL with NADP(H) [9].

Fluorescence spectroscopy was used to probe whether the mutated E126C residue may have altered the affinity of NfnL for NADP(H). Aromatic residues in the enzyme were excited at 285 nm and emission spectra were collected from 300 nm to 500 nm. Quenching of the protein fluorescence at 345 nm corresponded to increasing concentrations of NADP(H), as shown in Figure 3. The relative extent of quenching began to reach a plateau at NADP(H) concentrations above 40 µM, indicative of a saturation of binding sites via a specific binding interaction. NfnL-E126C did not demonstrate a difference in affinity for NADP^+^ versus NADPH, and fitting of the data to a quadratic equation for tight binding revealed a *K*_D_ of 2.6 ± 0.6 µM for binding to NADP(H). This value is comparable to the *K*_D_ of 3.0 ± 0.4 µM previously determined for NfnL-WT with NADP(H), which also bound the oxidized and reduced forms of this substrate with equal affinity [9]. Therefore, mutation of the site-differentiated ligand to the proximal FeS cluster has not significantly altered the affinity of NfnL for NADP(H), eliminating altered binding of the substrate tasked with instigating bifurcation as an explanation for the observed differences in the dye-based steady-state assays with NfnL-E126C.

### 3.4. Full-Bifurcation and Full-Confurcation Activity Assays

**Figure 4:**
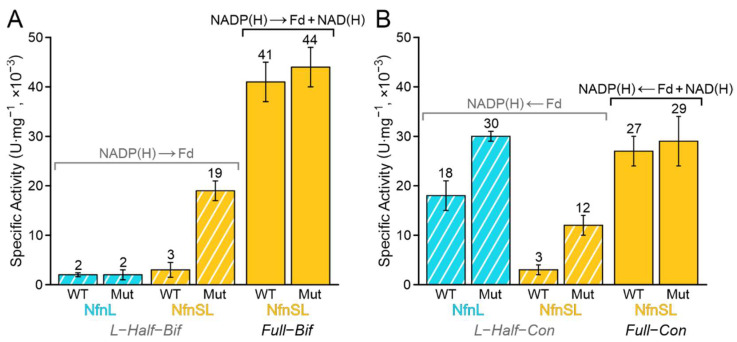
Half- (striped) and full-bifurcation (solid) (**A**) and confurcation (**B**) activity with NfnL-WT and NfnL-E126C in the absence (teal) and presence (gold) of NfnS. While there was no significant difference in activity between NfnSL-WT and NfnSL-E126C for the full-bif or full-con reactions, the half-reactions revealed more variability. In the half-bif reactions, NfnL-WT, NfnL-E126C, and NfnSL-WT demonstrated minimal ability to reduce Fd. NfnSL-E126C, however, retained the ability to transfer electrons along the low-potential branch even in the absence of NAD^+^, though to a lesser extent than in the full-bif reactions. The half-con reactions displayed minimal reactivity with NfnSL-WT only, whereas NfnL-WT, NfnL-E126C, and NfnSL-E126C all demonstrated some ability to oxidize Fd, with isolated NfnL-E126C showing as much activity as both forms of NfnSL for full-confurcation.

Though the outcomes of dye-based steady-state experiments can be helpful in identifying broad differences in the ability of an enzyme to mediate reduction of a dye, these assays typically provide limited mechanistic insight into the observed changes. With this in mind, and with the goal of gaining an understanding into the consequences of altering the non-canonical ligand to the proximal FeS cluster, the activity of NfnSL-WT and NfnSL-E126C was assessed with the physiologically relevant substrates. Full-bifurcation (full-bif) assays probed oxidation of NADPH for the coupled reduction of NAD^+^ and oxidized Fd, whereas full-confurcation (full-con) conditions used the oxidation of NADH and reduced Fd to drive reduction of NADP^+^. Full-bif and full-con reactivities were not investigated with the isolated NfnL subunits, since the absence of the high-potential pathway (i.e., NfnS) eliminates the NAD(H) reaction. Reactions assessed in these assays are summarized in Figure 2 and results are shown in Figure 4. Activity was higher for the full-bif reactions relative to the full-con ones with both forms of NfnSL, but no significant difference was observed between NfnSL-WT and NfnSL-E126C for either full reaction direction.

### 3.5. Half-Bifurcation and Half-Confurcation Activity Assays

Since alterations to energy allocation between NfnSL and its products may be obscured in the presence of all substrates, reactivity was also explored through half-reactions using NfnL-WT and NfnL-E126C, both in the absence and presence of NfnS. Half-bifurcation (half-bif) reactions employed NADPH oxidation for Fd reduction, and half-confurcation (half-con) conditions assessed oxidation of Fd for reduction of NADP^+^ (Figure 2 and Figure 4). Half-bif and half-con reactions with NfnSL-WT demonstrated minimal reactivity, consistent with what has been detailed in the literature for NfnSL when all three substrates are not present [10,23,24,25]. The half-reactions with NfnSL-E126C, however, still demonstrated activity around 40–45% of the full reactions despite the omission of NAD(H). This apparent uncoupling of the high- and low-potential branches in NfnSL-E126C may implicate a role for the site-differentiated glutamate ligand in maintaining the fidelity of the reactions when both branches are present. Isolated NfnL-WT and NfnL-E126C showed only nominal half-bif activity, comparable to that of the NfnSL-WT half-reactions. Surprisingly, both NfnL-WT and NfnL-E126C showed some level of half-con activity, with the latter generating as much oxidized Fd as the full-con reactions and the former demonstrating roughly half as much functionality. 

### 3.6. Electron Distribution along the High-Potential Branch of Nfn

Optical spectroscopy was used to explore the electron distribution among NfnSL cofactors when limited equivalents of NADPH were provided, with particular focus on the high-potential catalytic branch. In contrast to the concerted two-electron reduction of L-FAD by NADPH, which results in little-to-no accumulation of the extremely unstable ASQ species [9,10], the S-FAD accessory flavin can accept a single electron to yield a stable NSQ intermediate [9,10]. Formation of the S-FAD NSQ can be monitored by a distinctive increase in absorbance around ~610 nm [9,10]. Data reported here (Figure 4) and in other sources [10,23,24,25] indicate that NfnSL-WT is unable to bifurcate electrons in the absence of any one of the three physiological substrates. Bifurcation requisitely precedes reduction of oxidized S-FAD to the NSQ state and as such, NSQ should not accumulate appreciably in the absence of NAD^+^ and/or oxidized Fd. Therefore, in the WT enzyme, primarily L-FAD should be reduced when NADPH is the sole substrate present, resulting in a decrease in oxidized flavin absorbance at 450 nm with minimal change at 610 nm. Because NfnSL-E126C appears to result in an uncoupling of the two branches, we hypothesized that electrons could transfer into the high-potential branch upon addition of NADPH. 

**Figure 5:**
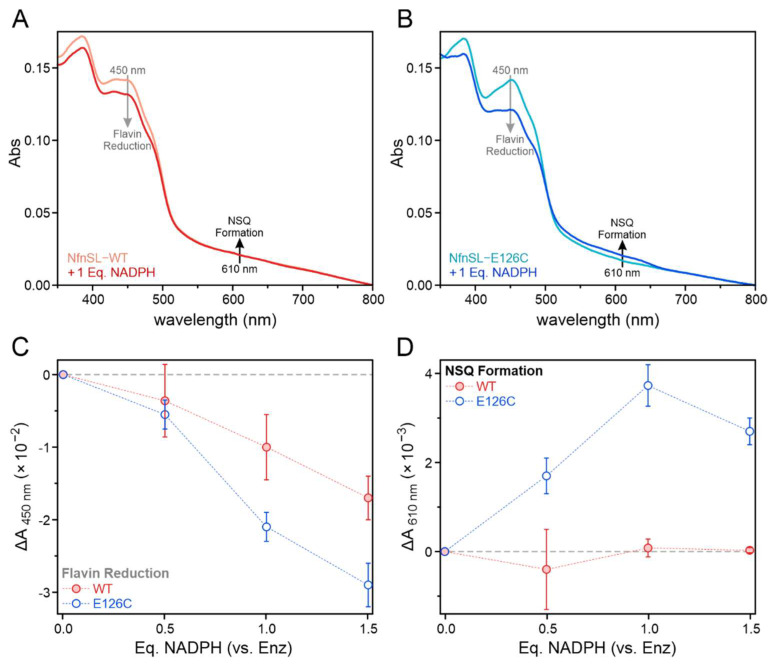
Electron distribution among the NfnSL flavins following addition of limiting equivalents (0, 5, 10, or 15 µM) of NADPH to 10 µM NfnSL-WT (red) or NfnSL-E126C (blue) was assessed using optical spectroscopy. Representative spectra of NfnSL-WT (**A**) and NfnSL-E126C (**B**), in the absence of the NAD^+^ and oxidized Fd substrates, revealed different extents of flavin reduction following addition of 1 Eq. of NADPH. Increased reduction of flavin at 450 nm (**C**; grey arrows in **A**,**B**) corresponded to higher NADPH concentrations with both forms of NfnSL, and the decrease in absorbance at this wavelength with NfnSL-E126C was approximately twice that of the WT protein. The NSQ intermediate of S-FAD, with absorbance around 610 nm (**D**; black arrows in **A**,**B**), was monitored to assess for electron transfer to S-FAD. NfnSL-E126C showed formation of the NSQ intermediate, whereas NfnSL-WT displayed little-to-no NSQ at the same concentrations of NADPH.

**Figure 6:**
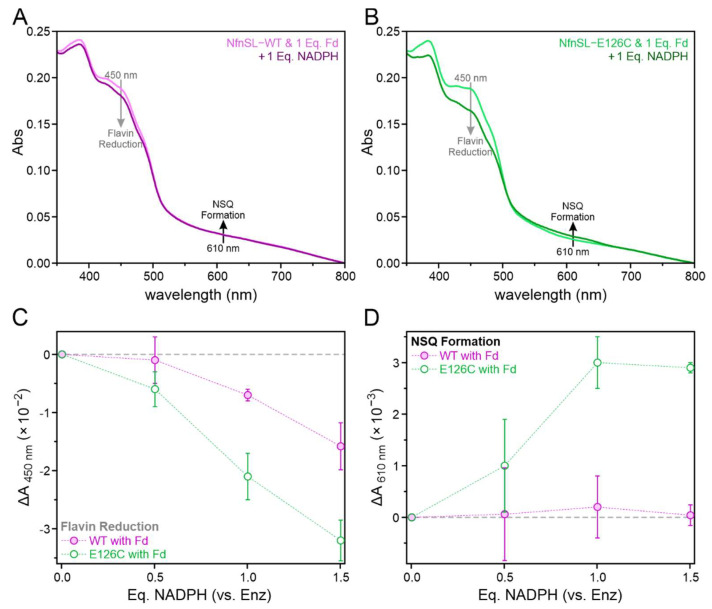
Varying concentrations (0, 0.5, 1, or 1.5 Eq.) of NADPH were incubated with 10 µM Fd and 10 µM NfnSL-WT (violet) or NfnSL-E126C (green) for analysis of the electron distribution along the NfnSL flavins via absorbance spectroscopy. Optical changes occurring upon addition of 10 µM of NADPH to Fd with either NfnSL-WT (violet) or NfnSL-E126C (green) are shown in (**A**,**B**), respectively. The decrease in absorbance at 450 nm (**C**; grey arrows in **A**,**B**) resulted from increasing equivalents of NADPH in both proteins, though NfnSL-E126C again exhibited roughly double the flavin reduction observed for WT. The increasing absorbance at 610 nm (**D**; black arrows in **A**,**B**) from S-FAD NSQ formation occurred more appreciably for NfnSL-E126C, but was again minimal for NfnSL-WT.

UV-visible absorption spectra were collected and analyzed for overall flavin reduction and NSQ formation following equilibration of NfnSL-WT or NfnSL-E126C with varying concentrations of NADPH, both in the absence (Figure 5) and presence (Figure 6) of oxidized Fd. Representative spectra (Figure 5A,B and Figure 6A,B) show changes in absorbance at 450 and 610 nm, which were then plotted as a function of NADPH equivalents for both NfnSL-WT and NfnSL-E126C (Figure 5C,D and Figure 6C,D). At 450 nm, NfnSL-E126C demonstrated roughly double the decrease in absorbance observed for WT at all non-zero NADPH concentrations, indicating a greater extent of flavin reduction regardless of the presence of Fd in the sample. The corresponding increase in absorbance at 610 nm with NfnSL-E126C, both with and without Fd, is consistent with NSQ formation at S-FAD. Minimal, if any, NSQ was formed in the WT samples. The altered electron distribution observed for NfnSL-E126C in the absence of the NAD^+^ substrate, relative to NfnSL-WT, indicates that an uncoupling of the high- and low-potential catalytic branches ultimately underlies the activity differences between the WT and E126C enzymes.

## 4. Discussion

To better understand the role of site-differentiated FeS ligands, specifically in the context of FBEB, we report a biochemical study in which the glutamate ligand that binds the proximal [4Fe-4S] in the low-potential pathway of NfnSL has been changed to a cysteine residue. Activity assays probing only the low-potential pathway demonstrated distinct differences between NfnL-WT and NfnL-E126C, both in the presence and absence of the NfnS subunit. Investigations into the binding of NfnL-E126C to NADP(H) resulted in a *K*_D_ value comparable to that of NfnL-WT for this substrate. Thus, the observed activity differences result from altered electron partitioning arising from the change in proximal FeS cluster ligation. Indeed, experiments analyzing the electron distribution along the high-potential branch cofactors showed more extensive electron accumulation on S-FAD in NfnSL-E126C than in NfnSL-WT. These studies have set a foundation to better understand how the protein environment controls the allocation of energy along the intra-enzyme pathways as well as within the chemical bonds of the NfnSL substrates.

Dye-linked assays show significant differences in steady-state parameters between the isolated NfnL-WT and NfnL-E126C subunits, with the *K*_M_ and catalytic efficiency of the latter exhibiting more similarity to results obtained with the NfnSL-WT and NfnSL-E126C heterodimers (which performed comparably to one another in this experiment). Differences in the ability of NfnL to reduce the dye in these assays does not owe to changes in the binding affinity of the NADPH substrate, as fluorescence-binding titrations revealed no alteration to the binding constant of NfnL-E126C for NADP(H) (Figure 3) relative to NfnL-WT [9]. Interaction between the dye molecule and the protein is likely non-specific and purely collisional, which is unlikely to change from alteration of the site-differentiated ligand in NfnL. This supports that something else, such as an altered electron distribution across the enzyme, may contribute to the observed changes in dye-linked activity.

In the confurcation direction, the generation of NADPH from reduced Fd is a thermodynamically favorable process according to the −320 [26,27] and ~ −400 mV [22] formal reduction potentials of NADP(H) and Fd, respectively. NfnSL-WT suppresses this pathway unless the reaction is coupled to the oxidation of NADH (Figure 4B) [10,23,24,25]. In the absence of NfnS, however, isolated NfnL-WT is able to produce NADPH from reduced Fd. Therefore, the physical connection of the two pathways appears to be a requirement for the energetic coupling of the two catalytic branches. While this has been postulated as a strict requirement for bifurcation [28,29,30,31], it has not been empirically demonstrated until now. NfnL-E126C also catalyzes the reduction of NADP^+^ from reduced Fd, but to a greater extent than NfnL-WT (Figure 4B, blue striped bars) with activity comparable to NfnSL-WT in the presence of all three substrates (Figure 4B, solid gold bars). Our data demonstrates that isolated NfnL, regardless of the specific ligation to the proximal FeS cluster, is biased towards the favorable oxidation of Fd. In fact, this particular half-reaction is essentially irreversible, with NfnL-E126C exhibiting an even larger bias for Fd oxidation than NfnL-WT (Figure 4A,B, blue striped bars). These results demonstrate that NfnL alone manages electron flux differently than NfnSL, and that this can be further perturbed by alterations to the unique site-differentiated FeS ligation of NfnL. 

Presumably, catalysis solely along the low-potential branch is limited in NfnSL-WT to inhibit short-circuit reactions. Short-circuit reactions are off-path electron transfer events that do not result in the conservation of energy. For example, a short-circuit occurs when an electron intended for the high-potential pathway is instead transferred to the low-potential pathway [8,28]. The isolated NfnL subunits appear to allow more electrons to “leak” through the low-potential branch when the reaction is overall thermodynamically favorable, as in the half-con reactions discussed above. In the presence of NfnS, NfnL-WT loses this capacity when only the low-potential branch substrates (reduced Fd and NADP^+^) are available. This is likely due to the energetic coupling between the two branches, regardless of which substrates are present. While NfnL-E126C with NfnS retains the ability to perform the favorable half-con reaction, it surprisingly also gains the ability to drive the unfavorable half-bif reaction (reduction of Fd from NADPH). This indicates that the mere presence of both pathways is not enough to entirely facilitate the energetic coupling at the heart of electron bifurcation, as these results directly implicate the site-differentiated ligation of NfnL as a contributing factor. 

The phenomenon of electron leakage between the catalytic branches in NfnSL-E126C does not appear to be confined to just the low-potential pathway. Assays assessing the accumulation of electrons at S-FAD of the high-potential branch, in the presence of either NADPH alone or both NADPH and oxidized Fd, showed an altered electron distribution in the NfnSL-E126C relative to the WT protein (Figure 5). Regardless of the presence of Fd, the former resulted in accumulation of the one-electron reduced NSQ intermediate, whereas the latter did not. Further, the decrease in oxidized flavin absorbance in NfnSL-E126C was roughly twice that of NfnSL-WT. This likely results from the two-electron reduction of a fraction of L-FAD in both enzymes, with additional reduction occurring at the S-FAD site in NfnSL-E126C. We hypothesize that S-FAD is reduced via the transfer of electrons from L-FAD along the high-potential branch in NfnSL-E126C due to an uncoupling of the two catalytic pathways. This demonstrates that the dysregulation of the electron distribution in NfnSL-E126C impacts the whole enzyme, not just the low-potential branch where the substitution occurs.

When NAD(H) is additionally included in the activity assays, the short-circuit that permits the leakage of electrons along the low-potential branch in NfnSL-E126C appears to be suppressed. This is demonstrated by the ability of NfnSL-E126C to achieve indistinguishable activity from NfnSL-WT in both the full-bif and full-con reactions (Figure 5A,B, solid gold bars). Allostery involving all three substrates has been shown for NfnSL-WT [10,23,30] and may contribute to this regulation, however, the specific molecular mechanisms have not been fully elucidated. Moreover, the experimental conditions may aid in the prevention of short-circuit reactions. An example of this is the imposed stoichiometry of the reaction set-up, since the substrates used in our activity assays were equal in concentration. Under these conditions, it is possible that the most energetically efficient pathways (i.e., the on-path reactions) could be favored, since no substrate is limiting, and therefore each branch would have a constant flux of electrons passing through it. This scenario would dictate that a short-circuit must compete with the on-path reaction, with the latter process likely several orders of magnitude faster [8,9,11]. This is consistent with theoretical investigations, which have shown that short-circuit reactions in NfnSL-WT are prevented by unfavorable thermodynamics and/or comparatively slow kinetics relative to the on-path routes [11]. 

Site-differentiated ligands found in various enzymatic systems have been shown to affect FeS cluster reduction potential, geometry, and electronic coupling, as well as to influence proton transfer capabilities and enzyme bias [12,13,32]. These types of modifications could impact electron transfer rates across the low-potential branch in NfnL-E126C and NfnSL-E126C. It is conceivable that the site-differentiated ligand modifies the thermodynamic landscape of Nfn in such a way that makes the short-circuit more competitive when the enzyme is not operating at full capacity, for example when one or more substrates are limiting [8,11]. Our work adds to the growing understanding of the roles non-cysteinyl FeS ligands play in different enzymatic contexts. In order to unambiguously assign the contributions of each of these possibilities, future work that includes a careful electron inventory and full thermodynamic assessment of the altered site-differentiated ligand needs to be explored in Nfn. These types of biophysical investigations will be necessary to fully deconvolute the interplay between short-circuit and on-path reactions in this system. 

An interesting feature of NfnSL-E126C is that electron leakage through the low-potential branch, owing to an increase in short-circuit reactions, is differentially controlled by the absence of one of the three substrates. The possibility to manipulate the intramolecular electron transfer events by controlling substrate concentrations and/or availability is an intriguing prospect presented by this work. By relaxing the strict pathway coupling afforded by the site-differentiated proximal FeS cluster, NfnSL-E126C would interface with the physiological redox pools in an unprecedented manner. Previous investigations of L-FAD thermodynamics have postulated that its uncharacteristically low reduction potential may act to restrict bifurcation activity to very specific cellular redox regimes [6]. It appears that the site-differentiated glutamate ligand imparts a similar constraint to link biochemical activity with intracellular conditions. Thus, our results reveal how nature has optimized NfnSL for integration into metabolic networks that rely on stringent modulation of NADP(H), NAD(H), and Fd levels. Furthering the knowledge of how these properties are coordinated will lead to a broader understanding of how NfnSL can influence flux through metabolic pathways. 

## 5. Conclusions

The biochemical impact and mechanistic roles of non-canonical FeS ligation are presently poorly understood, despite the incidence of site-differentiated ligands in many redox active enzymes. Though not ubiquitous in electron bifurcating systems, NfnSL homologs, complex III of the electron transport chain, and some bifurcating hydrogenases also contain non-cysteinyl FeS ligation [10,33,34,35]. The work presented here expands upon the known effects of site-differentiated ligation in redox active proteins while underscoring the breadth of mechanisms employed by bifurcating systems to ensure coupling of the two catalytic pathways. Further, the ability of a single substituted amino acid to perturb electron distribution across the NfnSL enzyme distinctly in response to varying substrate availabilities presents a remarkable opportunity for tailoring electron bifurcating enzymes for metabolic engineering endeavors. Altogether, our findings enrich the knowledge of components employed by electron bifurcating systems to achieve optimal energy conservation.

## Figures and Tables

**Table 1:** Results from benzyl-viologen-based steady-state experiments using the NADPH substrate with NfnL-WT or NfnL-E126C, with and without NfnS.

NfnL	±NfnS	*K*_M_ (mM)	*V*_max_ (mM·s^−1^)	*k*_cat_ (s^−1^)	*k*_cat_/*K*_M_ (mM^−1^·s^−1^)
WT	−	1.3 ± 0.1	0.05 ± 0.01	2.3 ± 0.3	1.7 ± 0.3
	+	0.37 ± 0.02	0.11 ± 0.02	5.7 ± 0.8	12 ± 3
E126C	−	0.30 ± 0.03	0.07 ± 0.01	3.5 ± 0.7	15 ± 2
	+	0.37 ± 0.01	0.12 ± 0.01	5.9 ± 0.6	16 ± 2

## Data Availability

The data presented in this study are available in this article.
